# Synthesis and Application in Polypropylene of a Novel of Phosphorus-Containing Intumescent Flame Retardant 

**DOI:** 10.3390/molecules15117593

**Published:** 2010-10-28

**Authors:** Jian-Dong Zuo, Shu-Mei Liu, Qi Sheng

**Affiliations:** 1 Shenzhen Key Laboratory of Special Functional Materials, College of Materials Science and Engineering, Shenzhen University, 518060 Shenzhen, China; 2 College of Materials Science and Engineering, South China University of Technology, 510640, Guangzhou, China; E-Mails: Shumeiliu@163.com (S.L.); mshengqi@yahoo.com.cn (Q.S.)

**Keywords:** triazine oligomer PMPT, intumescent flame retardant, phosphate-containing

## Abstract

A novel phosphorus-containing triazine oligomer poly(2-morpholinyl-4-penta-erythritol phosphate-1,3,5-triazine) (PMPT) was synthesized as a kind of tri-component intumescent flame retardant (IFR). The chemical structure of PMPT was characterized by FTIR, ^1^H-NMR and ^31^P-NMR, and the mechanical and flammability properties of FR-PP were measured. The FTIR results showed that the expected chemical reactions had happened at each step. The ^1^H-NMR and ^31^P-NMR spectra also agreed with the chemical structure of PMPT. The slight effect of PMPT on the mechanical properties of FR-PP suggested that PMPT and PP are compatible. The high limited oxygen index (LOI) values of FR-PP revealed that PMPT was an efficient IFR and there was the synergistic effect between PMPT and ammonium polyphosphate/ pentaerythritol (APP/PER).

## 1. Introduction

Intumescent Flame Retardant (IFR) systems have been used recently in flame retarded Polypropylene (FR-PP) as a kind of halogen-free flame retardant. Intumescent flame retardant compounds are usually composed of an acid source, a carbon source and a gas source, such as ammonium polyphosphate (APP), pentaerythritol (PER) and melamine [1]. However, the larger dosage of IFR compound needed and the poor compatibility with the polymer always impair the mechanical properties of PP.

A tri-component IFR is a new method which can reduce the amount of flame retardant and improve the thermal stability of the resulting FR-PP [2,3]. The main component of an intumenscent flame retardant is the element nitrogen, which has a good synergic flaming effect with phosphorus in the flame retarding [4,5]. It was found that triazines and their derivatives are good charring agents because of their abundant nitrogen and tertiary nitrogen structure [6,7]. Liu synthesized the phosphorus-containing triazine oligomer poly(2-piperazinylene-4-morpholino-1,3,5-triazine) (PPMT) and found that PPMT had a good effect in the delaying combustion of PP [8], but PPMT has no carbon atoms in the main molecular chain, so it has no carbon source and has to be used together with PER in order to achieve good flame retarding. In this paper, a novel phosphorus-containing triazine oligomer poly(2-morpholinyl-4-pentaerythritol phosphate-1,3,5-triazine) (PMPT) was synthesized and the chemical structure of the product was character by means of FTIR, ^1^H-NMR and ^31^P-NMR.

## 2. Results and Discussion

### 2.1. Reaction *mechanisms* (Scheme 1)

In tricyanogen chloride the stronger electron withdrawing group is the chlorine atom, which is a Lewis acid. There are lone pair electrons on the triethyl phosphate, which is a Lewis base. The reaction of tricyanogen chloride and triethyl phosphate can thus be regarded as a simple SN_2_ Lewis acid-base reaction. The net result is that the chlorine atom is easy to remove from the carbon atom and the yield of the acid-base reaction was improved 

**Scheme 1:**
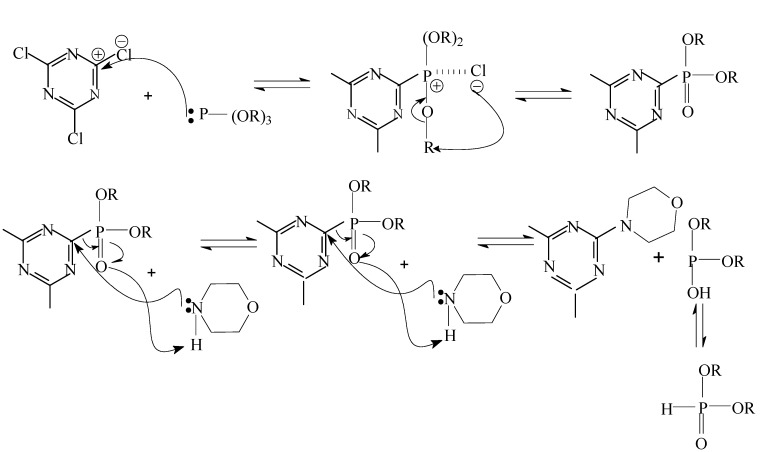
Reaction mechanism of PMPT.

In the second step the alkalinity of morpholine is greater than that of triethyl phosphate and the N atom has the stronger nucleophilicity on the morpholine, so it can attack the carbon atom with the positive charge close to the chlorine atom. The second reaction is therefore a nucleophilic substitution that can happen at room temperature. The reaction of compound **2** and pentaerythritol is a trasesterification. Dibutyl tin oxide as a neutral catalyst has no effect on the properties of the product and there is no need to separate it from the solution. Alcohol was continually evaporated from the solution in order to improve the yield.

### 2.2. FTIR of PMPT

**Figure 1:**
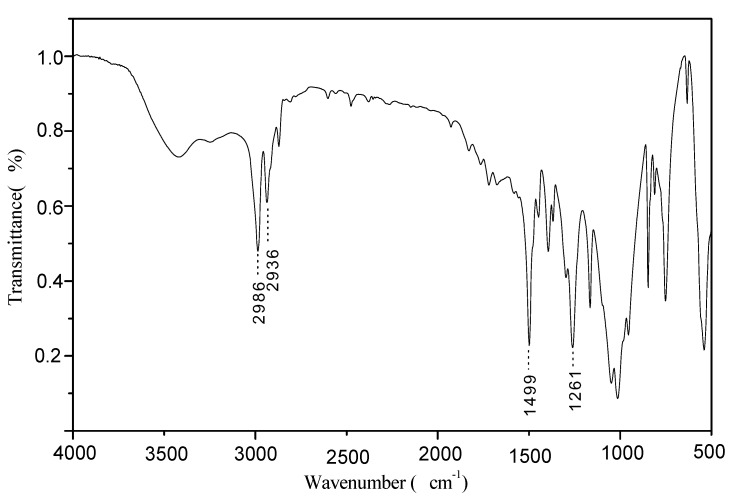
FTIR spectrum of compound **1.**

**Figure 2:**
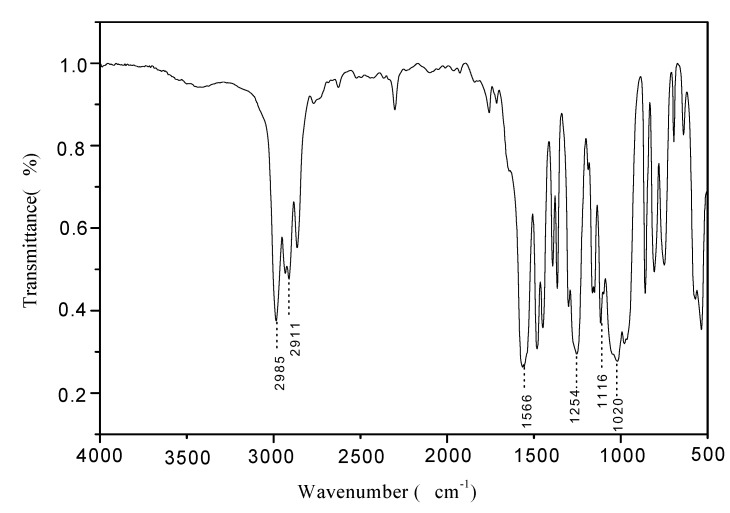
FTIR spectrum of compound **2.**

**Figure 3:**
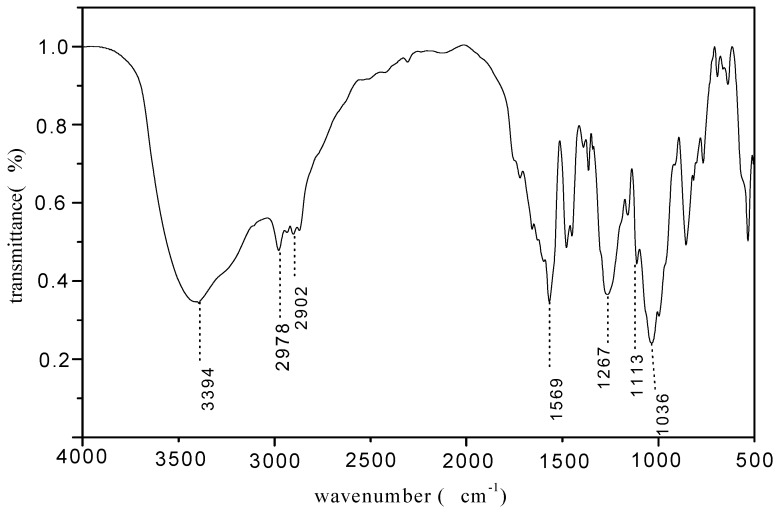
FTIR spectrum of phosphorus-containing triazine oligomer PMPT.

Figure 1, Figure 2 and Figure 3 show the FTIR spectra of compound **1**, compound **2** and PMPT, respectively. There are some specific absorption peaks for compound **1**. The bands at 2,986 cm^-1^ and 2,936 cm^-1^ were attributed to the stretching vibration of C–H in —CH_2_ and —CH_3_. The absorption at 1,499 cm^-1^ was assigned to the skeleton vibration of C=N in the triazine ring [9]. It is possible that the vibrational absorption of the triazine ring skeleton at 1,557 cm^-1^ shifts to the low frequency influenced by P=O group [10]. The band at 1,261 cm^-1^ is attributed to the stretching vibration of the P=O group, indicating that the expected chemical reaction between triethyl phosphite and tricyanogen chloride has indeed taken place. In Figure 2, the absorption signal at 1116 cm^-1^ was attributed to the asymmetric stretching vibration of the ether bond in the morpholinyl ring. The stretching vibration of P=O was slightly shifted to a lower frequency (1,254 cm^-1^).

In Figure 3, the absorption peak at 1,267 cm^-1^ still be assigned to the stretching vibration of the P=O group. The absorption peak of tertiary carbon bond can’t be clearly observed because of the overlap with the P=O group at about 1,245~1,180 cm^-1^ [11]. The appearance of the three P–O group absorption bands at 1,036 cm^-1^ showed that the phosphor-containing triazine oligomer had been generated. The strong band at 3,394 cm^-1^ was the absorption peak of the hydroxyl group. This ocurrs when pentaerythritol is the terminal group and the oligomer has the stronger water absorption.

### 2.2. ^1^H-NMR of PMPT

**Figure 4:**
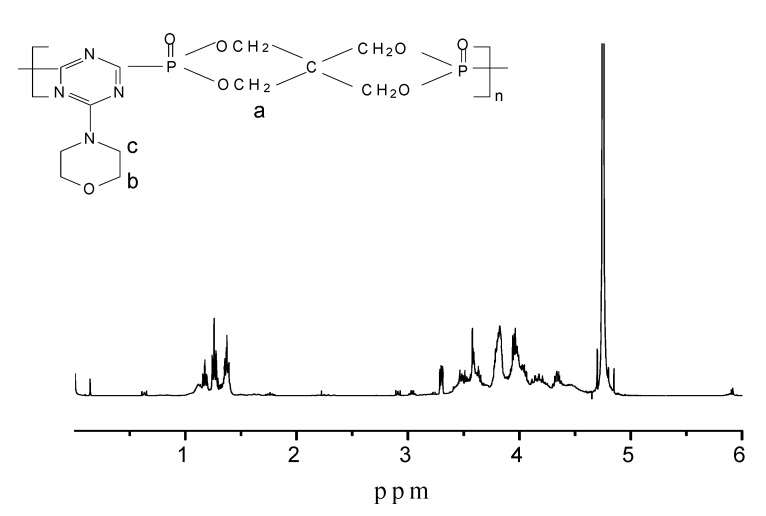
^1^H-NMR spectrum of phosphorus-containing triazine oligomer PMPT.

The ^1^H-NMR spectrum of PMPT is shown in Figure 4. The strong peak at δ = 4.75 ppm comes from the D_2_O solvent. The peak at δ = 3.81 was assigned to the superimposition of the methylene hydrogens a and b) joined with oxygen. The peak at δ = 3.35 was the methylene hydrogen joined with nitrogen (c). The peak at δ = 1.35 may be assigned to the methyl of product **2**, which didn’t completely react in step 3.

### 2.3. P NMR of PMPT

**Figure 5:**
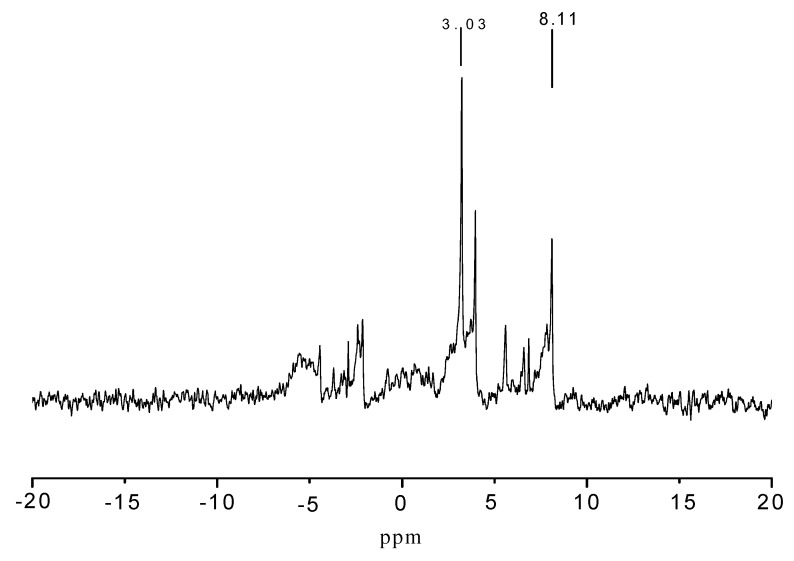
^31^P-NMR spectrum of phosphorus-containing triazine oligomer PMPT.

Figure 5 shows the ^31^P-NMR spectrum of PMPT. The atoms and groups surrounding the phosphorus atom generally affect its chemical shift, especially for the atoms in the four chemical bonds surrounding the central phosphorus atom [12].

**Figure 6:**
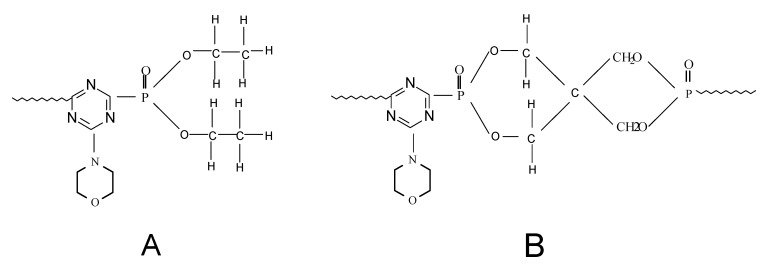
Phosphorus atoms in two different chemical environments.

Figure 6 shows the presence of two kinds of phosphorus atoms. The stronger absorption at 3.03 ppm was assigned to the shift of P=O group in product A [13]. The peak at 8.11 ppm was the chemical shift of phosphorus in material B. The donating electron ability of the groups decrease with the distance of the three chemical bonds surrounding the phosphor atom in material B, compared with that of phosphorus in material A, and the shielding effect of phosphorus atom on the external magnetic field becomes weaker, therefore the chemical shift of phosphorus in B is shifted to the low field. The ^1^H-NMR and ^31^P-NMR spectra are consistent with the chemical structures in Scheme 4.

### 2.4. Effect of PMPT and APP/PMPT on the flammability of FR-PP

Table 1 shows effects of PMPT and APP/PER/PMPT on the LOI values of FR-PP. The LOI values of FR-PP improved with the increasing of PMPT content. When the content of PMPT is 30 wt%, the LOI value of FR-PP increased about 59.7% compared with that of PP, while it can achieve UL-94 V-0 rating. The flammability of PMPT is better than PPMT made by the former procedure because PPMT hasn’t the sufficient carbon source and must be used together with APP, PER. Moreover the LOI value is slight higher than that of FR-PP with the same content of APP/PER. The LOI value can reach 29.4 after PMPT cooperates with APP/PER, which suggests that there is the synergistic effect between PMPT and APP/PER.

**Table 1:** Effect of PMPT and APP/PER/PMPT on the LOI.

Sample	LOI/%	UL94
Pure PP	18.1	HB
10%PMPT	23.4	V-2
30%PMPT	28.9	V-0
30%APP/PER (3/1)	27.8	V-0
30%APP/PER/PMPT (3/1/2)	29.4	V-0

### 2.5. Effect of PMPT on the mechanical property of FR-PP

Conventional IFRs always impair the mechanical properties of FR-PP because of the low thermal stability and the poor compatibility. Table 2 lists the mechanical properties of FR-PP with the different flame retardants. Though the mechanical properties decrease slightly with the increase of the PMPT content, the tensile strength and notched impact strength of FR-PP with 30% PMPT increases about 31% and 23% more than those of FR-PP with the same content of APP/PER. The main molecular chain of PMPT contains carbon atoms, therefore the compatibility of PMPT and PP is better than that of other IFRs.

**Table 2:** The mechanical properties of FR-PP with the different flame retardants.

Sample	Tensile strength (MPa)	Notched impact strength /J.m-1
Pure PP	33.1	41.2
10%PMPT	32.8	39.3
30%PMPT	28.8	33.8
30%APP/PER (3/1)	21.9	27.3
30%APP/PER/PMPT (3/1/2)	23.4	29.6

## 3. Experimental

### 3.1. General

IR spectra were recorded with a Vector-33 FTIR spectrometer using KBr pellets. ^1^H-NMR and ^1^^3^P- NMR spectra (D_2_O as solvent) were recorded on a Bruker Avance Digital spectrometer (operating at 400 MHz for ^1^H and ^13^P) . The limited oxygen index (LOI) values were measured on a FTT oxygen index meter (England Fire Testing Technology Co., Ltd) with sheet dimensions of 130×6.5×3mm according to ISO 4589-1984. The vertical flammability test is measured according to UL-94 test using 150×13×3 mm^3^ specimens.

### 3.2. Materials

Triethyl phosphite was supplied by Shandong Jincheng Pharmaceutical & Chemical Co., Ltd. Tricyanogen chloride was obtained from Sigma-Aldrich Co. (St Louis, MO, USA). Analytically pure pentaerythritol was purchased from Shanghai Kefeng Chemical Reagents Co., Ltd. Morpholine was obtained from Shanghai Nanxiang Reagents Co., Ltd. The other reagents were all analytical reagents. Ammonium polyphosphate (APP) was obtained from Shandong Linqin Fuyuan Chemical Co., Ltd. Polypropylene was a commercially available grain with a 0.8 g/10 min melt flow rate and was purchased from Sinopec Yanshan Chemical Corporation..

### 3.3. Methods

**Scheme 2:**
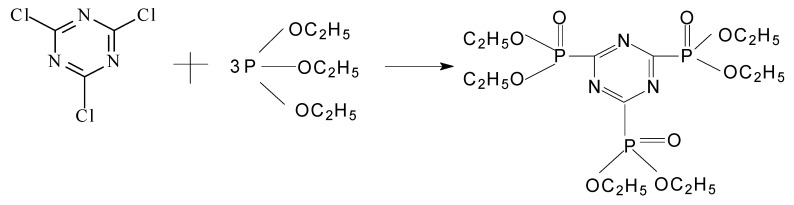
Synthesis of compound **1.**

First, in a 1 L four-necked glass flask, equipped with a stirrer, thermometer, pressure equalizing addition funnel and cooling bath, tricyanogen chloride (46.1 g) and methylbenzene (250 mL) were added. Then triethyl phosphite (124.5 g) was added in 1 h while the mixture was stirred. The reaction mixture was then heated up to 70 ^o^C for 6 h until no more gas was generated. Yellow crystals could be obtained after the methyl benzene was evaporated from the mixture and it was cooled down to room-temperature. The crystals were filtered and recrystallized from ethyl ether. The white crystals of 2,4,6- tri(diethyl phosphate)-1,3,5-triazine (**1**) weighed 105.4 g (yield: 86.1%). The preparation of compound **1** was shown in Scheme 2.

**Scheme 3:**
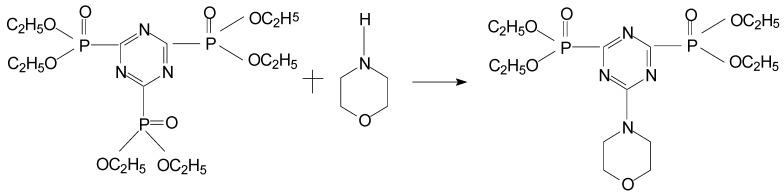
Synthesis of compound **2**.

Second, in a 1 L four-necked glass flask, equipped with a stirrer, thermometer, pressure equalizing addition funnel and cooling bath, compound **1 (**97.8 g) and absolute alcohol (500 mL) were added. Morpholine (17.4 g) was then added and the mixture stirred for about 3 h at room temperature. Then the alcohol was evaporated from the mixture and the residue were washed by *n*-hexane and ethyl ether compound (300 mL, volume ratio: 1:4) and the filtrate was concentrated at room temperature. The white crystalline product, 2-morpholinyl-4,6-di(diethyl phosphate)-1,3,5-triazine (**2**) weighed 81.1 g (yield: 92.5%). The reaction is shown in Scheme 3.

Third, in a 500 mL three-necked glass flask, equipped with a stirrer, thermometer and heating bath, compound **2** (43.8 g), pentaerythritol (13.6 g), dibutyl tin oxide catalyst (1.0 g) and N,N-dimethyl-formamide (DMF, 200 mL) were added.

**Scheme 4:**
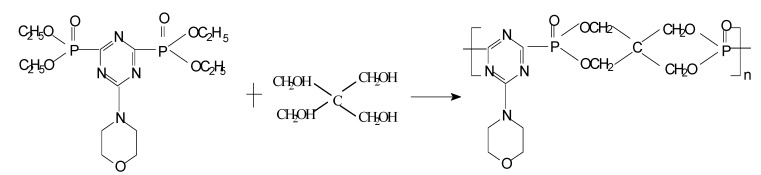
Synthesis of PMPT.

The mixture was stirred for 24 h at 130 ^o^C and the alcohol was evaporated continually. After this, a yellow solid was obtained after the DMF was distilled off. The white crystalline phosphorus-containing triazine oligomer poly(2-morpholinyl-4-pentaerythritol phosphate-1,3,5-triazine) (PMPT, **3**) weighed 49.1 g (yield: 93.6%) was the after the yellow solid was recrystallized from tetrahydrofuran and washed with alcohol.

## 4. Conclusions

The phosphorus-containing triazine oligomer PMPT was synthesized by a three step chemical reaction. The band at 1,261 cm^-1^ in the FTIR and attributed to the P=O group stretching vibration indicated that the chemical reaction between triethyl phosphite and tricyanogen chloride had occurred. The absorption signal at 1,116 cm^-1^ was attributed to the asymmetric stretching vibration of the ether link on the morpholinyl ring. ^1^H-NMR and ^31^P-NMR spectra further confirmed the chemical structure of PMPT. The slight effect of PMPT on the mechanical properties of FR-PP suggested that the compatibility of PMPT and PP is better than other IFRs. The high LOI values of FR-PP revealed that PMPT is an efficient IFR and there is the synergistic effect between PMPT and APP/PER**.**
